# A one-step procedure to probe the viscoelastic properties of cells by Atomic Force Microscopy

**DOI:** 10.1038/s41598-018-32704-8

**Published:** 2018-09-27

**Authors:** Ya Hua Chim, Louise M. Mason, Nicola Rath, Michael F. Olson, Manlio Tassieri, Huabing Yin

**Affiliations:** 1 Division of Biomedical Engineering, School of Engineering, University of Glasgow, Glasgow, G12 8LT UK; 2 Cancer Research UK Beatson Institute, Garscube Estate, Switchback Road, Glasgow, G61 1BD UK

## Abstract

The increasingly recognised importance of viscoelastic properties of cells in pathological conditions requires rapid development of advanced cell microrheology technologies. Here, we present a novel Atomic Force Microscopy (AFM)-microrheology (AFM^2^) method for measuring the viscoelastic properties in living cells, over a wide range of continuous frequencies (0.005 Hz ~ 200 Hz), from a simple stress-relaxation nanoindentation. Experimental data were directly analysed without the need for pre-conceived viscoelastic models. We show the method had an excellent agreement with conventional oscillatory bulk-rheology measurements in gels, opening a new avenue for viscoelastic characterisation of soft matter using minute quantity of materials (or cells). Using this capability, we investigate the viscoelastic responses of cells in association with cancer cell invasive activity modulated by two important molecular regulators (i.e. mutation of the p53 gene and Rho kinase activity). The analysis of elastic (*G*′(*ω*)) and viscous (*G*″(*ω*)) moduli of living cells has led to the discovery of a characteristic transitions of the loss tangent (*G*″(*ω*)/*G*′(*ω*)) in the low frequency range (0.005 Hz ~ 0.1 Hz) that is indicative of the capability for cell restructuring of F-actin network. Our method is ready to be implemented in conventional AFMs, providing a simple yet powerful tool for measuring the viscoelastic properties of living cells.

## Introduction

The importance of cell mechanical properties has become increasingly recognised in the study of cell function and fates. The cell cytoskeleton, modulated by either intracellular or extracellular stimuli, can control cell growth, differentiation, and death^1^. Deformation of the cell cytoskeleton is strongly linked with the onset and progression of human diseases^2^. To investigate these mechanotransduction processes, in-depth understanding of the mechanical properties of cells at both the molecular and cellular levels is essential.

Since a cell cytoskeleton consists of a network of filamentous proteins (e.g. actin, microtubules, and intermediate filaments) with high water content, cells can store (solid-like) and dissipate (liquid-like) energy according to the rate at which the stimulus is applied - a typical viscoelastic behaviour^3^. However, how these cells respond to stimuli from the molecular to cellular levels is poorly understood^4^, since intracellular movements of small molecules and large aggregates occur over broad time scales^5,6^.

Various technologies for quantifying the viscoelastic properties of cells have been developed over the past decades^7^, such as optical tweezers^8^, mechanical micropipette aspiration^9^, magnetic tweezing cytometry^5,8^ and atomic force microscopy (AFM)^10^. In comparison to some of these techniques, AFM offers a significant advantage in that it can directly measure living cells in their physiological conditions with force and spatial resolution of the order of pico-Newtons and nanometres, respectively. To date, viscoelasticity measurements using AFM can be broadly classified as either oscillatory frequency measurements or time-dependent indentation (i.e. stress-relaxation) measurements^11^. Oscillatory frequency measurements are the most commonly used, however, measurements in liquid are beset with hydrodynamic forces that are strongly influenced by the experimental settings^12,13^. In addition, the deformation of the material is measured in response to a sinusoidal stress at a given frequency, with a range of accessible frequencies defined by the capacity of the used instrument, typically between 0.1 Hz to 200 Hz for the majority of commercial AFMs^14,15^, and up to 100 kHz for the recently developed high-speed AFMs^16,17^. In contrast, time-dependent AFM mechanical tests uses a quasi-stationary stress-relaxation measurement, but requires fitting the force-indentation curves with a preconceived phenomenological model to determine the ‘pseudo’ material parameters describing the viscoelastic response of cells^18,19^. In general, these parameters are dependent on the experimental setup (e.g. holding time) and are prone to fitting errors associated with the chosen models and the estimation of unknown parameters^10^.

Here, we present a novel AFM-microrheology method for educing the linear viscoelastic properties of complex materials and living cells, over five continuous decades of frequency (i.e. 0.005 Hz ~ 200 Hz), from a simple stress-relaxation (nanoindentation) measurement using a commercial AFM instrument. The experimental data have been directly analysed without the need of interpreting the experimental data with preconceived viscoelastic models. The method has been validated against conventional oscillatory bulk-rheology measurements on a range of complex materials, and further exploited to investigate the viscoelastic responses of cells in association with cancer cell invasive activity. To this end, we have chosen two important molecular regulators that are closely associated with cancer metastasis, namely mutation of the p53 gene^20^ and Rho kinase (ROCK) activity^21^, and have evaluated how these molecular interventions modulate cell mechanics to facilitate cancer invasion. Mouse pancreatic ductal adenocarcinomas (PDAC) cell lines have been selected for this study. Characteristic mechanical properties that link molecular regulation, cell morphology and cell migration models have been discovered, providing new insights into cancer cell invasion. The experimental approach is ready to be implemented in conventional AFMs, providing a simple yet powerful tool to study mechanical properties of soft matter in general.

## Results

### Establish the AFM-Microrheology (AFM^2^) method for measuring the materials’ continuous frequency spectrum

**Figure 1:**
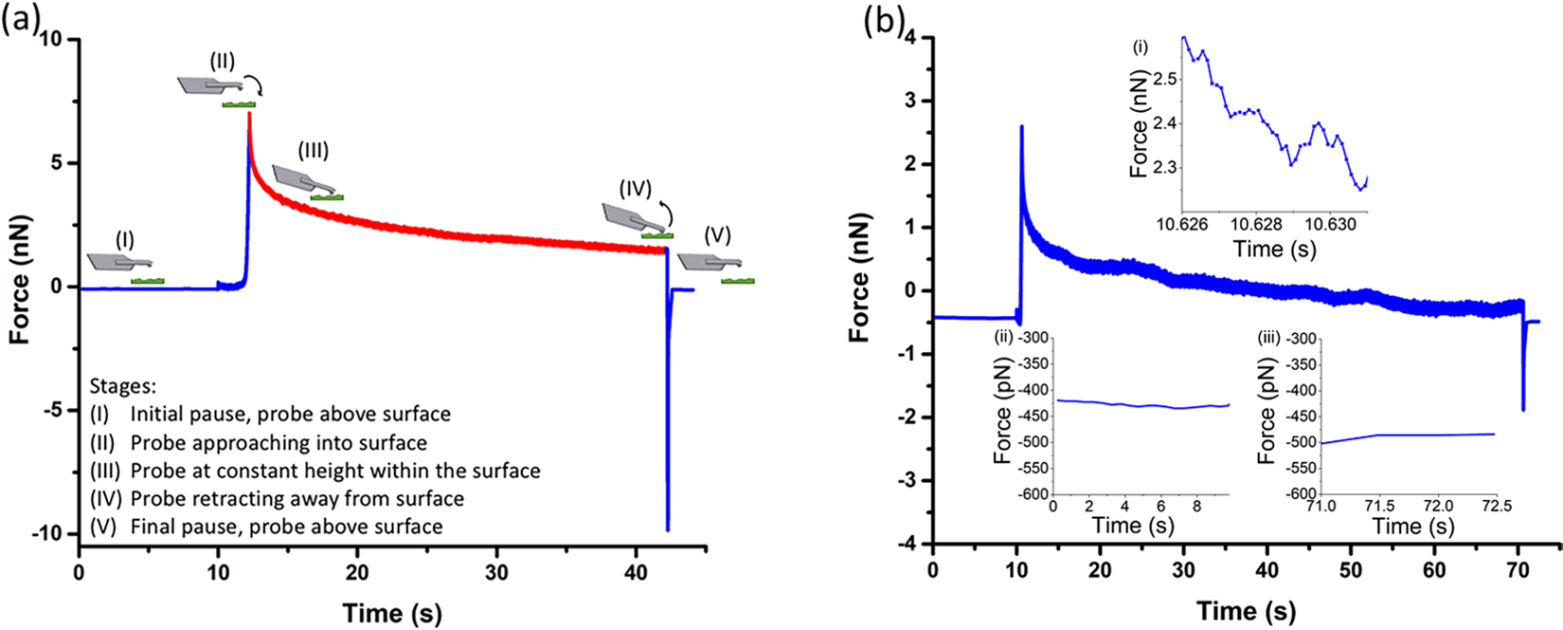
(**a**) Procedure for stress-relaxation nanoindentation measurement. The probed cantilever was equilibrated above the sample surface for 10 s (I), then indented into the sample to the predefined indentation depth (II), maintained at the constant height for a certain period of time while recording force deflection (III), retracted away from the surface (IV) and returned to its original position (V). The closed-loop feedback control is used to control the Z Piezo movement. The deflection force and indentation data in the Stage (III) were collected at a high sampling rate (e.g. 8 kHz or 16 kHz). (**b**) Representative raw stress-relaxation data from a cell measured using an ARROW-TL1 cantilever with an attached 4.7 μm silica bead, spring constant 0.05 N m^−1^. Graph inserts show the force values during the (i) first 5 ms of the relaxation measurement, used to estimate the viscous drag force, (ii) initial and (iii) final pause segments of the cantilever while stationary above the substrate. This indicates negligible deflection drift within the system throughout the measurement.

Stress-relaxation experiments were carried out by indenting a sample with a step displacement and recording the resultant force response over time (Fig. 1a,b). Prior to the stress-relaxation measurement, an indentation depth of <10% of the sample thickness was determined by single indentation measurement, allowing the implementation of a modified Hertzian model for a spherical indenter^22^. A spherical indenter not only provides a clearly defined geometry but also prevents penetration into cells. To satisfy the assumptions of the Hertz model and minimize substrate effect, a 400 nm indentation depth above the nuclei area was used^23^. This also reduces variability within a cell, allowing comparable study of different cell populations^24^. The deflection force and indentation data were collected at a high sampling rate, and analysed to derive viscoelastic properties of the sample, as described below.

Analysis of the stress-relaxation data in our AFM^2^ approach is underpinned by the same principles underlying the new rheological tool, “i-Rheo”^25^, which allows the evaluation of the material’s linear viscoelastic properties over the widest range of experimentally accessible frequencies from a simple time-dependent step-strain measurement. Key to this approach is the Fourier transform of raw experimental data^26,27^ describing both the time-dependent stress and strain functions. In the case of AFM, where experiments provide force *F*(*t*) and indentation *δ*(*t*) measurements, the constitutive equation for linear viscoelasticity is based on the principle that the effects of sequential changes in indentation are additive^28,29^:

1 $$F(t)={\int }_{-\infty }^{t}G(t-\tau )\dot{{\rm{\Lambda }}}(\delta (\tau ))d\tau $$

where *G*(*t*) is the material’s shear relaxation modulus and $\dot{{\rm{\Lambda }}}(\delta (t))$ is the time derivative of Λ(*δ*(*t*)). The latter is the material’s instantaneous deformation introduced by the Hertz’s model, which for a rigid spherical indenter on an incompressible linearly elastic half-space:

2 $$F=E\frac{4}{3}\frac{{R}^{1/2}}{(1-{\nu }^{2})}{\delta }^{3/2}=G\frac{8}{3}\frac{{R}^{1/2}}{(1-\nu )}{\delta }^{3/2}$$

where *E* is the Young’s modulus, *R* is the radius of the spherical probe and *v* is the Poisson ratio (commonly assumed to be 0.5). For the second equality of Equation 2 it has been assumed that *E* = 2 *G*(1 + *v*)^30^. Moreover from Equation 2 it is possible to educe the functionality of Λ(*δ*(*t*)) in terms of *δ*(*t*):$\,{\rm{\Lambda }}(\delta (t))=\frac{8}{3}\frac{{R}^{1/2}}{(1-\nu )}{\delta }^{\frac{3}{2}}(t)$; where the probe radius *R* and *v* are constant once the experiment is designed.

Equation 1 is a convolution integral between the two time-dependent functions G(t) and Λ(δ(t)) and its Fourier transform results in the product of these functions Fourier transformed. Therefore, in the frequency domain Equation 1 can be written as:

3 $${G}^{\ast }(\omega )\equiv i\omega \hat{G}(\omega )=\frac{\hat{F}(\omega )}{\hat{{\rm{\Lambda }}}(\omega )\,}=G^{\prime} (\omega )+iG^{\prime\prime} (\omega )$$

where $\hat{G}(\omega )$, $\hat{F}(\omega )$ and $\hat{{\rm{\Lambda }}}(\omega )$ are the Fourier transforms of *G*(*t*), *F*(*t*) and Λ(*δ*(*t*)), respectively. G^*^(*ω*) is the material complex shear modulus, which is a complex number whose real *G*′(*ω*) and imaginary *G*″(*ω*) components provide information on the elastic and viscous nature of the material, respectively. They are commonly named as the material’s storage and loss moduli, respectively.

To implement these analytical procedures, a Labview executable was developed to derive the storage (*G*′(*ω*)) and loss (*G*″(*ω*)) moduli directly from the input of raw force – indentation data (Supplementary Fig. S2). In principle, the range of accessible frequencies is dictated by the extremes of the experimental time window [*t*_*min*_, *t*_*max*_]: i.e. *ω*_*max*_ = 1/*t*_*min*_ (rad/s) and *ω*_*min*_ = 1/*t*_*max*_ (rad/s)^25^, where *t*_*min*_ is the time of the first measured point after *t* = 0 (i.e. the inverse of the sampling rate), and *t*_*max*_ is equal to the duration of the experiment. However, because of the uncertainties in the initial contact point and the nonlinear deformation of the material during the indentation process^31^, only force relaxation data were analysed (i.e. *t* = 0 is set to the point when the indentation reaches the set value and the force starts to relax, as indicated by the red line in Fig. 1a)^32^. In the case of a 30 s relaxation holding time, *ω*_*min*_ = 0.0333 rad/s (i.e. ${f}_{min}=\frac{{\omega }_{min}}{2\pi }\approx 0.005\,{\rm{Hz}}$). It should be noted that although the majority of commercial AFMs can reach 10′s kHz sampling rate, their Piezo performance become frequency-dependent above 100′s Hz. This becomes the upper limit of the experimentally accessible frequencies. Since the transfer function of the Z Piezo used in this study shows minimal frequency independence up to ~300 Hz (Supplementary Fig. S3), we set 200 Hz as the upper frequency limit in this study. Moreover, the highest deflection rate of the cantilever occurs within the first 5 ms of the relaxation process, with a value of circa 1 µm/s for a typical cell measurement (calculated from a 5 nm deflection at 5 ms, Fig. 1b(i)). Therefore, by assuming a viscous coefficient of ~10 pN·s/µm^13,33^, the viscous drag force on the cantilever is estimated to be of the order of 10 pN; which is negligible compared to 289 pN change due to relaxation within the same time window.

The probe approaching speed is a well-known factor influencing the elasticity measurements that use the Hertzian model to interpret the indentation data^12^. To evaluate its influence on AFM^2^ analysis, a range of indentation speeds (i.e. 3, 10, 30, 70 and 100 μm/s) were examined with a 100 kPa commercial gel. Over-shoot of the defined load force (i.e. defined indentation depth) was observed for indentation speeds >30 μm/s (Supplementary Fig. S4a). Therefore, a loading rate of 10 μm/s was chosen for the rest of the investigations. The viscoelastic property of the 100 kPa gel obtained at this speed showed good agreement with that from the bulk-rheology measurement (Supplementary Fig. S4b).

### Validation of the AFM^2^ method using a range of complex materials

**Figure 2:**
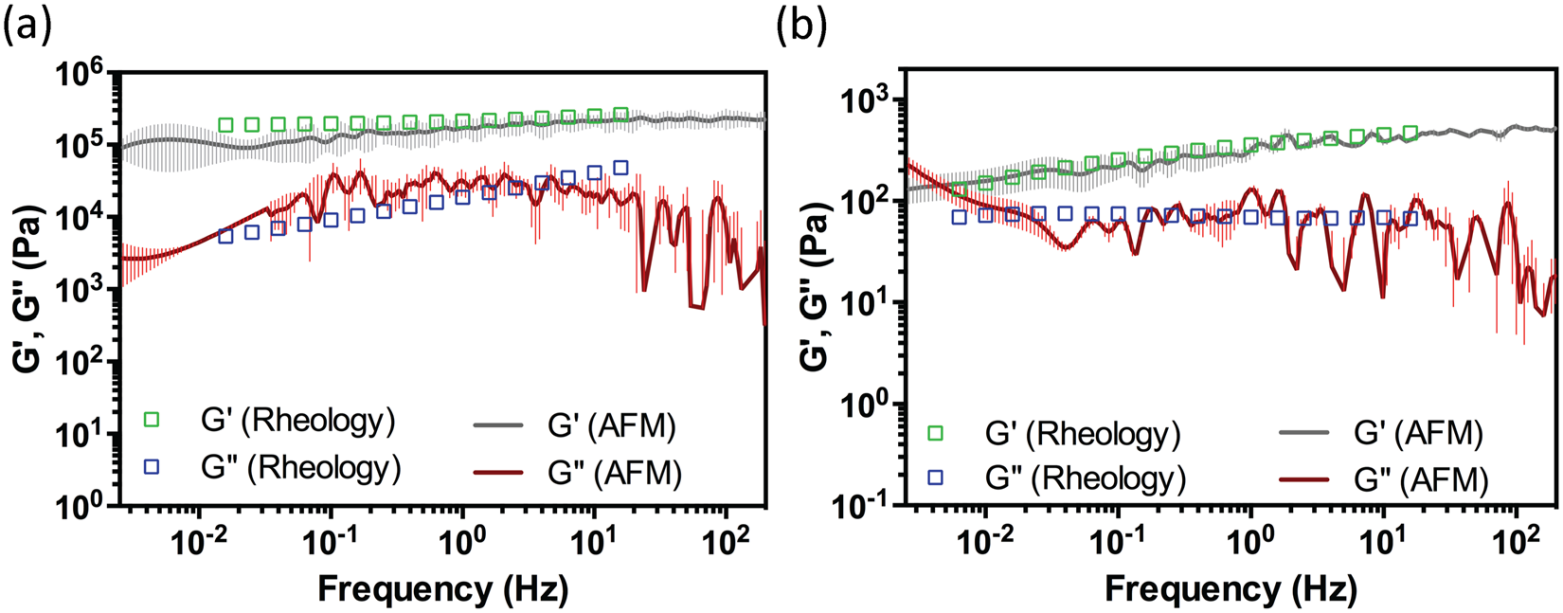
Comparison between the materials’ viscoelastic moduli (*G*′*(ω)* and *G*′′*(ω)*) measured by a conventional bulk-rheology rheometer (open symbols) and the AFM^2^ method (solid line); (**a**) 20:1 polydimethylsiloxane (PDMS) gel, using a FORT-TL cantilever with an attached 20 μm silica bead. (**b**) 5% polyacrylamide gel-like solution, using an ARROW-TL cantilever with an attached 4.7 μm silica bead.

Quantitative comparisons between the AFM^2^ approach and conventional linear oscillatory measurements were carried out on significantly different complex materials, including freshly made 20:1 PDMS gel and a 5% polyacrylamide gel-like solution (PAAM). Discrete *G*′(*ω*) and *G*″(*ω*) moduli values were obtained from shear oscillatory bulk-rheology measurements over a frequency range of 0.1 < ω < 100 rad/s; whereas, a continuous spectrum of the moduli was determined using the AFM^2^ method over five frequency decades (Fig. 2). Within the common frequency range, the viscoelastic response of the investigated materials showed very good agreement between the two methodologies; both in terms of absolute values and frequency dependence (Fig. 2). In particular, a crossover frequency between the *G*′(*ω*) and *G*″(*ω*) moduli of the PAAM solution (Fig. 2b) (a common feature for a viscoelastic fluid) was observed via the AFM^2^ approach. These results show that the AFM^2^ method gives the opportunity to measure the materials’ linear viscoelastic properties over a wide range of frequencies from the analysis of a simple step-indentation AFM measurement. The good agreement with conventional bulk-rheology measurements provides the required confidence for the adoption of this method to investigate the viscoelastic properties of living cells (under the assumption of a *quasi* time-invariant system). This provides new insights on relationships between the mechanical properties of living cells and their pathological states.

### Interrogating viscoelastic responses of cells in a “single-step” measurement

Although AFM has been widely used for evaluating mechanical properties of cells, the majority of work is limited to the elasticity (i.e. the Young’s modulus) of cells. The novel AFM^2^ approach offers, for the first time, a simple procedure to probe both the viscous and the elastic properties of living cells in their physiological conditions, over a broad range of continuous frequencies. Here, the AFM^2^ method was exploited to understand the importance of cells’ viscoelastic properties in cancer cell invasion.

We selected two recently observed contributors involved in enhanced cancer invasion, namely mutation of the p53 tumor suppressor gene^20^ and ROCK overexpression^21,34^, and investigated how cellular viscoelastic responses were modulated by these two mechanisms and whether viscoelastic responses plays a role in the increased invasion activity. Since cells have to change their shape during migration, we firstly evaluated the viscoelastic responses of cells with different shapes that were formed by culturing cells on micropatterned surfaces.

#### The effect of morphology on the viscoelastic behaviour of cells

**Figure 3:**
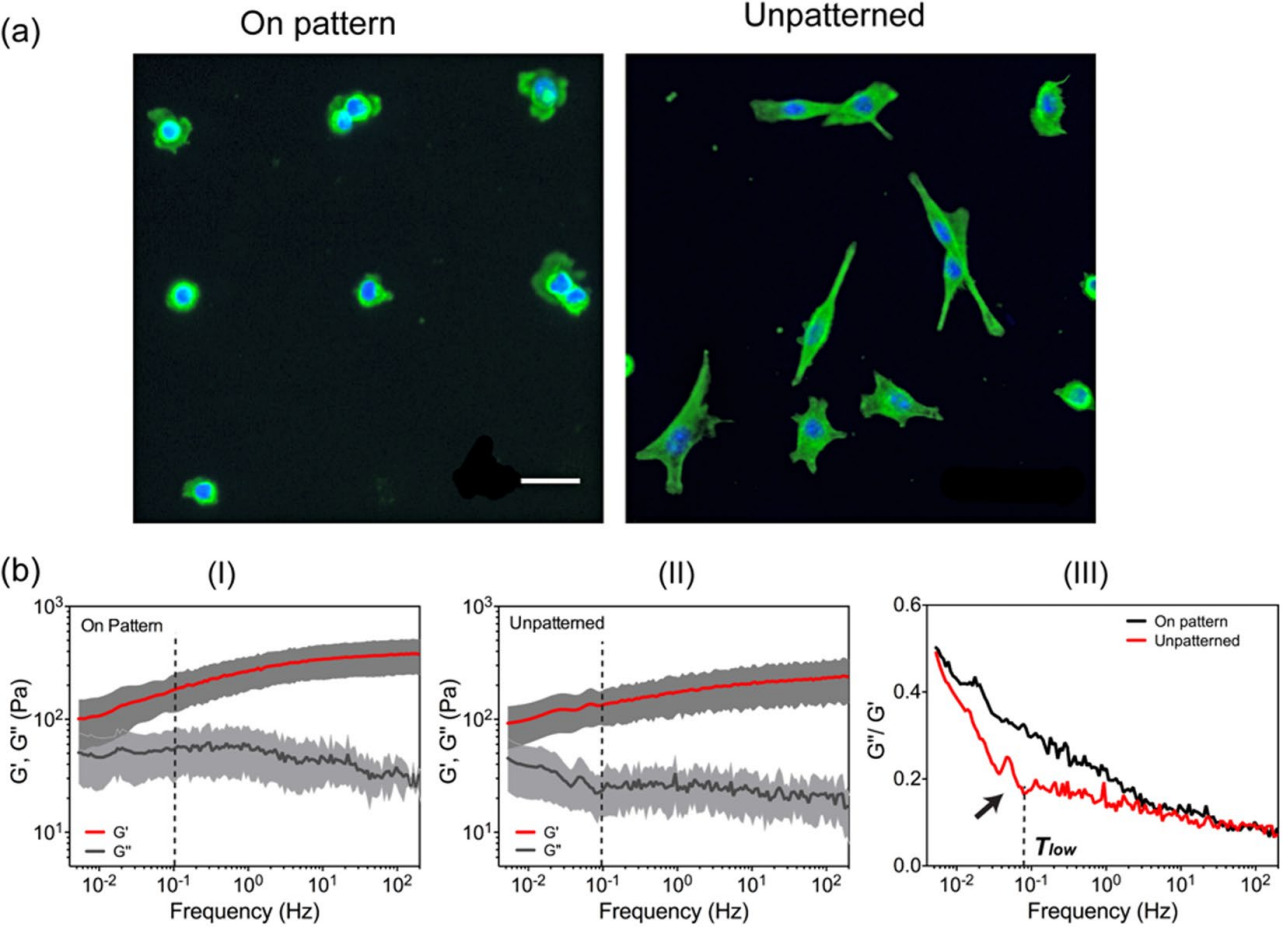
The effect of morphology on cell viscoelasticity. (**a**) Immunofluorescence images of PDAC P53^R172H^ cells on patterned and unpatterned surfaces. Scale bar 20 μm. Notice that only single cells spread to the boundaries of the pattern were analysed. (**b**) The storage modulus (*G*′(*ω*)), the loss modulus (*G*″(*ω*)) and loss tangent (*G*″(*ω*)/*G*′(*ω*)) of cells on patterned and non-patterned substrates. All cell measurements were carried out using an ARROW-TL1 cantilever with an attached 4.7 μm silica bead. 50 randomly selected cells were measured in each condition. Data were given as the mean values (central line) and standard deviations (shade area) in (I) & (II). The mean values were used to calculate the loss tangent. The dotted lines in (I) & (II) indicate different zones as described in the text. The black arrow in (III) shows a transition point.

Morphological changes can result from biochemical modulations^35^. Therefore, we first evaluated the influence of cell shape on their viscoelastic properties. The micropatterning technique was used as a simple physical means to regulate cell shape as well as restrict cell movement^36–40^. Mutant PDAC p53^R172H^ cells were cultured on a micropatterned substrate with an array of 20 μm adhesive circles, giving rise to round cell shapes on the patterns (Fig. 3a). The F-actin network in the round cells was diffuse and mainly concentrated at the periphery forming the cell cortex. There was no obvious stress fibre formation inside the cytoplasm (Fig. 3a). In contrast, mutant PDAC p53^R172H^ cells cultured on an unpatterned substrate were elongated with an intensive network of interwoven F-actin filaments and stress fibres across the cytoplasm (Fig. 3a).

Using the AFM^2^ method, the viscous and elastic moduli of both round and spread cells over five decades of continuous frequencies were obtained (Figs 1b and 3b). For each population, 50 cells were measured. D’Agostino & Pearson omnibus normality test of the distribution of their *G*′(*ω*) or *G*″(*ω*) values was carried out at each frequency. In the majority of cases, *p* values are above 0.05, suggesting a normal distribution. For example, at 0.1 Hz, the viscoelastic moduli *G*′(*ω*) and *G*″(*ω*) of patterned cells show *p* values of 0.0930 and 0.1741, respectively. Therefore, average values of cells were given. For frequencies between 1 Hz and 200 Hz, both cell shapes showed *G*′(*ω*) decreasing with frequency, with a weak power-law dependence, *G*′(*ω*) ∝ *ω*^*β*^, where *β* is found to be ~0.045 ± 0.008 by linear fitting of *logG*′(*ω*) versus log(*ω*) (with a R-squared value of 0.954); When f <1 Hz, an increase of the power-law dependence of *G*′(*ω*) was observed for both cell shapes, with *β* equal to 0.13 ± 0.01 for the spread cells (with a R-squared value of 0.984) and 0.17 ± 0.003 for the round cells (with a R-squared value of 0.98). It is worth noting that the *β* values for *G*′(*ω*) of both cell shapes in this frequency range are in good agreement with many other cell lines reported previously, where values of the power law exponent range between 0.12–0.25^8,14,31^.

However, in the case of *G*″(*ω*), we didn’t observe common power-law dependence as reported previously^16,23,31,41–43^. For frequencies between 1 Hz and 200 Hz, *G*″(*ω*) hardly varied for the spread cells, whereas *G*″(*ω*) of the round cells showed a slightly negative power law with a *β* value of −0.119 ± 0.007 (and a R-squared value of 0.829). For frequencies <1 Hz, *G*″(*ω*) of the round cells remained almost constant until the lowest frequency, whereas *G*″(*ω*) of the spread cells on non-patterned substrates were insensitive to frequency until f ≈ 0.08 Hz and increased sharply with decreasing frequency below this value. These results are clearly different from previous studies with passive/active microrheology^5,8,44^ and AFM-based oscillatory measurements^14,16,23,45^. They not only reflect cell intrinsic properties (e.g. cytoskeleton structure, oncogene expression) but also are likely affected by the measurement parameters (e.g. the indentation depth) as detailed in the discussion.

The loss tangents (*G*″(*ω*)/*G*′(*ω*)) provide a measure of the relative contribution of the viscous and the elastic components. It is often used as an indicator of the presence, position and relative magnitude of the transitions between different states. A small loss tangent (*G*″(*ω*)/*G*′(*ω*) < 1) reflects a dominant solid-like elastic behaviour, which enables cells to maintain their shape in response to deformation. In contrast, a large loss tangent (*G*″(*ω*)/*G*′(*ω*)>1) indicates a liquid-like behaviour. The loss tangents of cells on patterned and on unpatterned surfaces were both sensitive to frequency (Fig. 3b-III), illustrating dynamic changes in cell behaviour. However, they also contain significant differences as detailed below:

1. For frequency between 1 Hz and 200 Hz, *G*″(*ω*)/*G*′(*ω*) of both cell shapes increased rapidly with decreasing frequency, indicating a transition to viscoelastic behaviour. The loss tangent of the rounded cells on patterns was consistently larger than that of the cells on unpatterned substrates, suggesting a more deformable nature (i.e. fluid-like behaviour) of the round cells. This is not surprising because they lack intensive F-actin network and stress fiber formation (Fig. 3a).
2. When f ≲ 1 Hz, interestingly, a characteristic transition (denoted as ***T***_***low***_) occurred at f ≈ 0.08 Hz (i.e. ~12 s from the start of relaxation) (Fig. 3b-III, black arrow), below which a steep increase in the gradient of the loss tangent with decreasing frequency occurred. However, this phenomenon was only observed for the spread cells on unpatterned substrates. In contrast, the loss tangent of the cells on patterns continuously increased at the same gradient and reached 0.6 at the lowest frequency, indicative of a viscoelastic liquid-like behaviour.

It is worth noting, because of geometrical restrictions, cells on patterns were prevented from changing shape and have limited adhesion sites on the adhesive patterns, whereas their counterparts on unpatterned substrates could reorganise their cytoskeleton and move freely^36,37^. This indicates that cells on patterns have limited capability of reorganising their cytoskeleton, dynamically restructuring F-actin networks and modulating interactions with substrates. These limitations may lead to the absence of the characteristic transition (***T***_***low***_) in the cells on patterns. Similar phenomena to this characteristic transition have been observed for unbinding of transient cross-linked actin networks as a consequence of initiation of cytoskeleton restructuring to actively dissipate energy^46,47^. Taken together, the existence of the ***T***_***low***_ transition, therefore, can serve as an indicator of a cell’s capability for dynamic reorganisation of its cytoskeleton.

#### The role of the p53 gene on mechanical properties of cells and its association with cancer invasion

**Figure 4:**
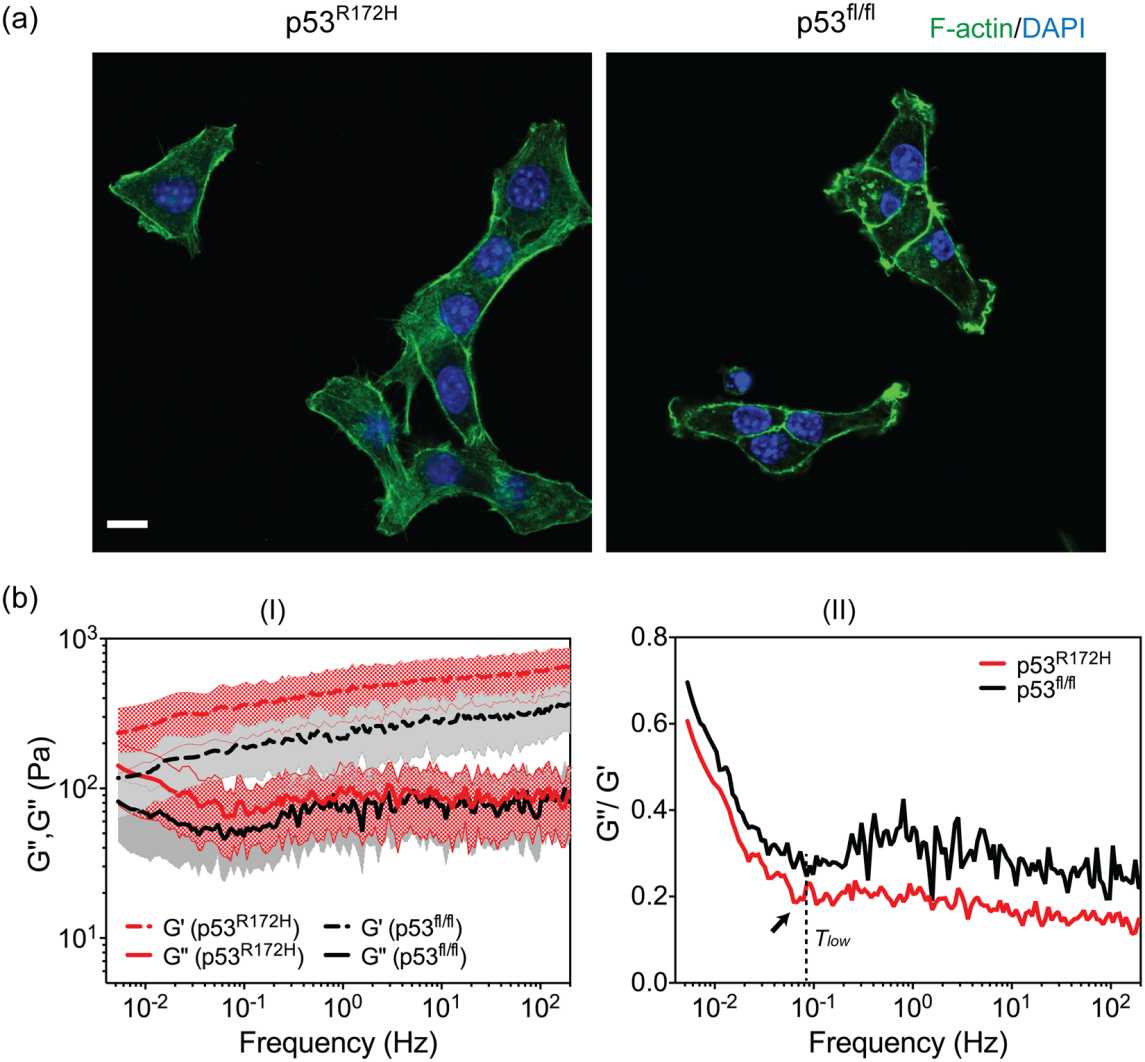
(**a**) Immunofluorescence images of PDAC P53^R172H^ and PDAC p53^fl/fl^ cells on non-patterned surfaces, scale bar 10 μm. (**b**) Complex moduli (*G*′(*ω*), *G*″(*ω*)) and loss tangent (*G*″(*ω*)/*G*′(*ω*)) of both cell lines (n = 50 in each cell line). The dashed line and black arrow bar indicate the ***T***_***low***_ transition point. The mean values and standard deviations were given for the complex moduli. The mean values were used to calculate loss tangent.

*TP53* is a tumour suppressor gene and is often mutated in human pancreatic cancer through missense mutations^48^. Mutant PDAC p53^R172H^ cells exhibit invasive activity and a pro-metastatic function, whereas p53 deleted PDAC p53^fl/fl^ cells are non-invasive^20^. In 2D culture, mutant PDAC p53^R172H^ cells were stretched either as individual cells or in groups (Supplementary Fig. S5). The majority of cells contained extensive networks of F-actin filaments and stress fibers across the cytoplasm (Fig. 4a, left). In contrast, PDAC p53^fl/fl^ cells formed clusters. Individual cells within colonies adopted round shapes with an average diameter of ~13 μm (Supplementary Fig. S5) and contained strong peripheral F-actin microfilaments at the boundaries between cells (Fig. 4a, right). However, weak, scattered F-actin filaments were occasionally observed within the cytoplasm.

Over the five frequency decades, *G*′(*ω*) and *G*″(*ω*) of both cell lines showed the same frequency dependence (Fig. 4b-I), and *G*′(*ω*) of the PDAC p53^R172H^ cells were 40–50% higher than the PDAC p53^fl/fl^ cells (p < 0.01). In the case of *G*″(*ω*), no differences were observed except in the lower frequency end (*f* < ~0.1 Hz) where *G*″(*ω*) of the invasive PDAC p53^R172H^ cells were slightly higher (Fig. 4b-II). These results suggest that the PDAC p53^R172H^ cells are stiffer, which is in good agreement with their more extensive stress fibre formation - the major contributor to cellular stiffness^49^.

Interestingly, despite the different morphology of both cell lines (i.e. spread versus round shape when cultured on conventional petri-dish), their *G*″(*ω*)/*G*′(*ω*) showed similar frequency dependence over the whole frequency range, and importantly, ***T***_***low***_ occurred at a similar frequency (*f* ≈ 0.08 Hz) (Fig. 4b-III). This behaviour for the PDAC p53^fl/fl^ cells is in striking contrast to the round PDAC p53^R172H^ cells on patterns (Fig. 3b-III), despite their similar morphology. As discussed above, the absence of ***T***_***low***_ for the patterned PDAC p53^R172H^ cells is associated with their limited capability of reorganising their cytoskeleton and restricted movement^36,37^. The presence of ***T***_***low***_ in PDAC p53^fl/fl^ cells shows that individual cells in a cluster were not limited by their neighbouring cells, and maintain similar levels of restructuring activities as the freely moving PDAC p53^R172H^ cells.

#### Effects of ROCK signalling on viscoelasticity plays a role in cancer cell invasion

Rho-associated kinase (ROCK) is a well-known effector protein in the regulation of actin cytoskeleton organisation^50^. A plethora of studies show ROCK signalling is involved in tumour cell motility and metastasis^21^. It was found that increased ROCK activity in PDAC cells facilitates invasive PDAC tumour growth^34^. However, how such elevated activity affects cell mechanical behaviour, and whether it contributes to invasive tumour growth, is not clear. Here, the non-invasive PDAC p53^fl/fl^ cells were modified to express ROCK1 and ROCK2 estrogen-receptor (ER) fusion proteins (denoted as ROCK1:ER and ROCK2:ER cells respectively), which can be conditionally activated in the presence of 4-hydroxytamoxifen (4HT). Cells tagged with non-catalytic GFP:ER fusion proteins (denoted as GFP:ER cells) were used as a control. All the modified cells lines showed a similar, cluster-like morphology as the parental PDAC p53^fl/fl^ cells.

**Figure 5:**
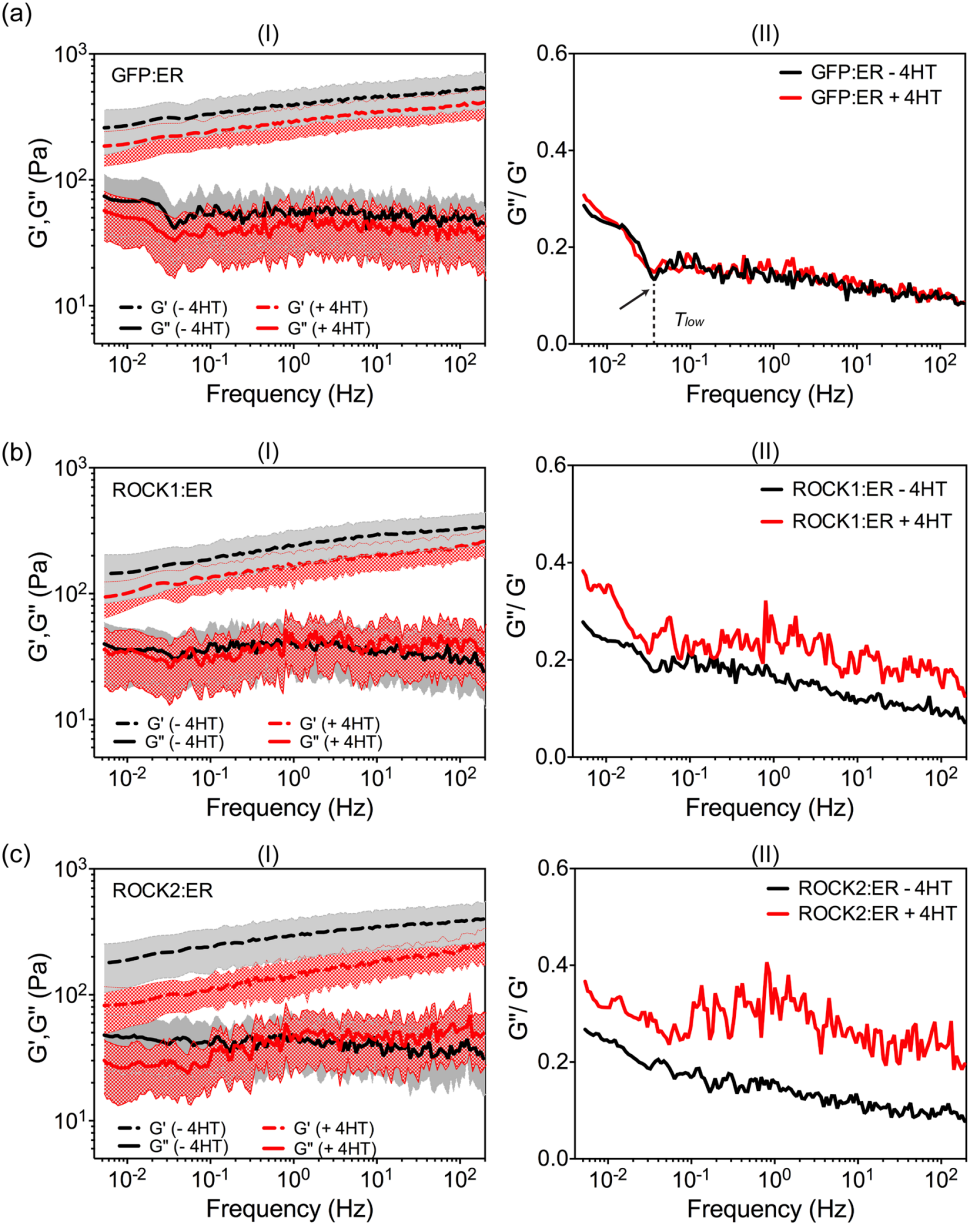
Complex moduli (*G*′(*ω*), *G*″(*ω*)) and loss tangent (*G*″(*ω*)/*G*′(*ω*)) for (a) GFP:ER, (**b**) ROCK1:ER, and (**c**) ROCK2:ER cells with and without 4HT in the medium (n = 50 for each treatment). Mean values and standard deviations were given for the complex moduli. The mean values were used to calculate loss tangent.

In comparison to the parental PDAC p53^fl/fl^ cells, it was found that the retroviral transduction and selection of cells expressing non-catalytic GFP:ER significantly increased *G*′(*ω*) (P < 0.0001) but not *G*″(*ω*) (Supplementary Fig. S6a). Interestingly, this stiffening effect did not influence the existence of the ***T***_***low***_ transition in the loss tangent curve (Fig. 5a–II, arrow), suggesting the expression of non-catalytic GFP:ER fusion proteins does not substantially reduce active cytoskeleton restructuring. However, the ***T***_***low***_ transition occurred at much lower frequency (i.e. *f* ≈ 0.03 Hz) as a consequence of the stiffening effect. In contrast, expression of ROCK1 and ROCK2 ER fusions in the parental PDAC p53^fl/fl^ cells did not cause obvious changes to *G*′(*ω*) but significantly reduced *G*″(*ω*) values (Supplementary Fig. S6b,c). The ***T***_***low***_ transition on the loss tangent curve was not obvious for ROCK1:ER cells and hardly observable for ROCK2:ER cells (Fig. 5b,c-II). These observations suggest that the existence of ***T***_***low***_ transition is affected more substantially by the variations of viscous properties. The expression of ROCK:ER fusion proteins had a negligible effect on elastic properties, but made cells more fluid (i.e. less viscous) and reduced the overall capability of dynamic cytoskeleton restructuring. The phenomena matches well with the actions of ROCK in stabilizing F-actin and promoting filament bundling, both of which can reduce dynamic cytoskeleton reorganisation.

Upon the addition of 4HT, *G*′(*ω*) of both ROCK1:ER and ROCK2:ER cells reduced across the five frequency decades (p < 0.0001) (Fig. 5). This reduction effect was more significant in ROCK2:ER cells. No significant changes were observed on the *G*″(*ω*) values for both cell lines. However, both cells after 4HT treatment showed a weak power law dependence for *G*″(*ω*) (Fig. 5b,c-I, the red dotted lines). This change was more apparent in ROCK2:ER cells, where *β* is found to be ~0.07 over the whole frequency range (and a R-squared value of 0.733). This was in apparent contrast to the plateau value observed prior to the treatment, indicating a substantial change in cell cytoskeleton. In addition, the resultant higher loss tangent values also suggested that cells become more liquid-like (Fig. 5b,c-II). In contrast, the addition of 4HT did not have obvious effects on GFP:ER cells, as shown in their completely overlapped loss tangent curves (Fig. 5a-II), further confirming that GFP:ER fusion proteins are non-responsive to 4HT.

**Figure 6:**
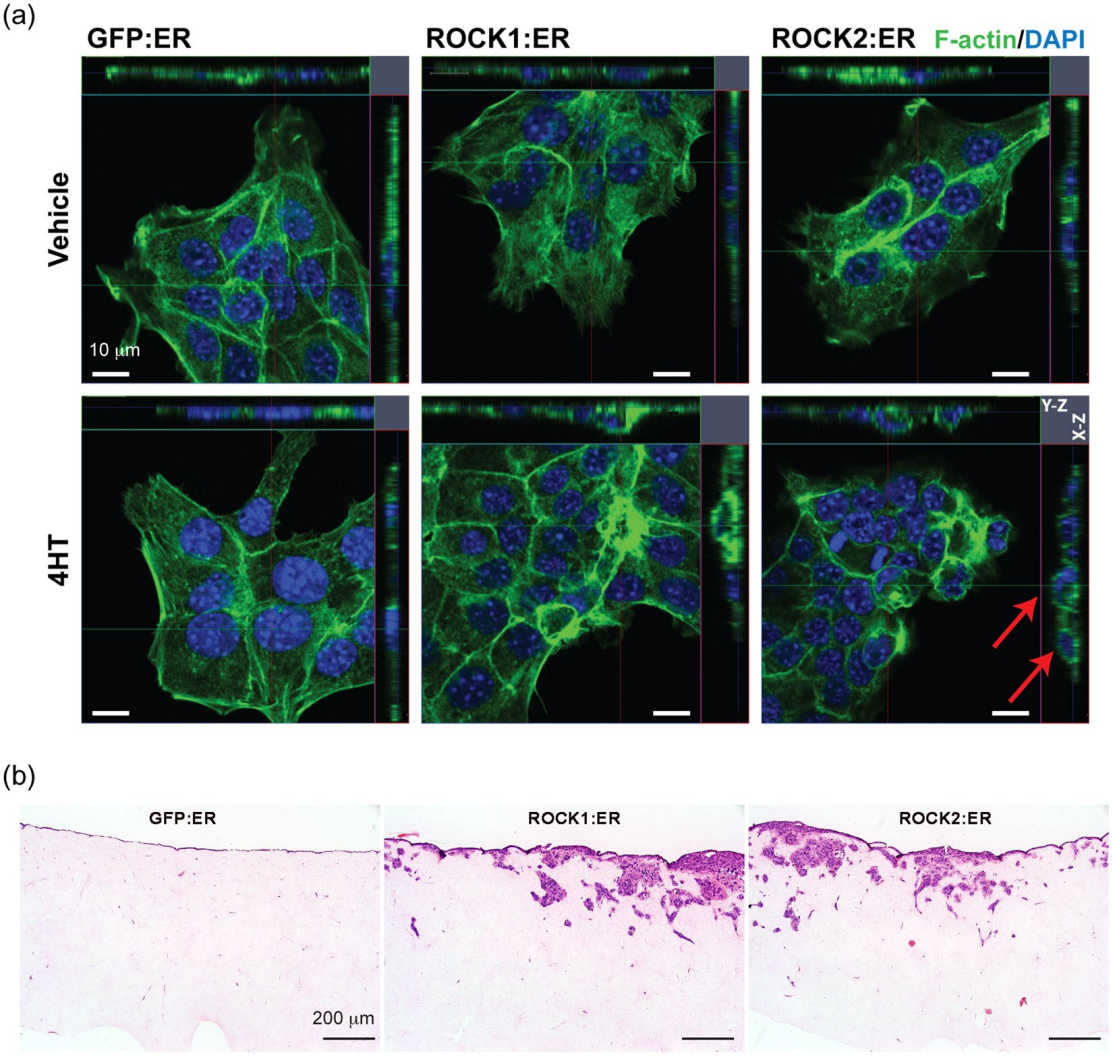
(**a**) Immunofluorescence images of GFP:ER, ROCK1:ER and ROCK2:ER expressing cells with and without 4HT activation. (**b**) H&E stained sections of cell invasion into collagen matrix after 8 days. All cell lines were treated with 1 μM 4HT. Arrows indicate the F-actin rich structure.

Immunofluorescence imaging further corroborated the effect of 4HT (Fig. 6a). While the morphology and F-actin stress fibers remained largely unchanged with and without 4HT in GFP cells, 4HT treatment reduced internal F-actin bundles for both ROCK:ER cells, with more significant effects on ROCK2:ER cells. These structural changes account for the observed reduction in elastic modulus as shown previously^49^. Interestingly, upon 4HT treatment, both ROCK:ER cells rounded up (e.g. the height of cells increased from ~3.5 μm to ~8 μm) and showed a thicker actin cytoskeleton over the cell nuclei area (see Fig. 6 arrows). It is well-established that actin filament network plays a dominant role in the frequency behaviour of *G*″(*ω*)^4,51^, thus such a change may account for the weak power law dependence of *G*″(*ω*) for ROCK:ER cells treated with 4HT. Furthermore, their peripheral F-actin ring became thinner, indicating reduced spreading and adhesion to the substrate, increased cellular contraction, and reduced intercellular tension. In comparison to GFP:ER cells, the 4HT activation substantially enhanced ROCK:ER cell invasion into 3D collagen matrices (Fig. 6b), where clusters of cells migrated as a collective group. It is easy to conceive that reduced interaction with external surfaces and more liquid-like behaviour of individual members would facilitate coherent movement of the group. Taken together, it indicates that active ROCK modulates structures and mechanical properties that facilitate collective migration.

## Discussions

### A generic, microscopic method for measuring soft matter

We introduce a novel procedure that quantifies the elastic (*G*′(*ω*)) and the viscous (*G*″(*ω*)) moduli of living cells over five decades of continuous frequencies from a single-step relaxation AFM nanoindentation. Compared to previous AFM-based methods^11,14,17,45^, our approach is simple and does not involve data fitting with preconceived viscoelastic models. It can be readily implemented in commercial instruments with no additional apparatus, making it a generic, readily implemented approach for AFM-based technologies.

As illustrated with a range of complex materials, the frequency range measured via this microscopic method significantly extends beyond the limits of common bulk-rheology methods. Importantly, within the frequency ranges allowed by the instruments, the viscoelastic moduli measured by the two methods are in very good agreement. This offers a distinct advantage since values from microrheology and macrorheological measurements often differ^46^. As such, this method opens a new avenue for viscoelastic characterisation of a wide range of biomaterials with minute quantity and in physiological environments.

### A simple approach for cell microrheology over a broad range of continuous frequencies

Cell viscoelastic properties can be measured via a number of methods. However, depending on the experimental techniques, different cellular structures are probed, and thus reveal mechanical properties of different subcellular structures. For example, magnetic twisting cytometer measures the motion of magnetic beads randomly attached to the cytoskeleton via integrins, which do not discriminate mechanical responses based on cell shape or specific subcellular location^5^. Similarly, various formats of tracking microrheology (e.g. optical tweezers) monitor the motion of microbeads injected into cell cytoplasm, which reflects local intracellular rheological properties. However, in these approaches, the bead probes are randomly distributed within or on a cell, resulting in uncontrolled spatial resolution. AFM-based microrheology overcomes the uncertainties of probe location and allows associating the measured mechanical responses with underlying cellular structures. This capability is very desirable for probing living cells because of highly heterogeneous mechanical properties of individual cells^14,45^. However, conventional stress-relaxation AFM microrheology does not give the samples’ frequency-dependent viscoelastic properties. Active oscillatory AFM-microrheology relies on successive measurements of individual frequencies, which can be time consuming for a wide range of frequency. Indeed, the majority of micro-rheology methods reported on cells’ viscoelastic properties are limited to frequencies >0.01 Hz^8^.

Our new AFM^2^ method offers a fast and simple method to obtain viscoelastic behaviour of cells over a broad, continuous frequency range and at precisely defined locations from a single-step stress-relaxation measurement. The capability of revealing respective elastic (*G*′(*ω*)) and viscous (*G*″(*ω*)) responses of living cells to imposed stresses, allows for association of underlying molecular mechanics with macroscopic mechanical behaviours. Although the current work only demonstrated the measurement of viscoelastic moduli over the continuous frequencies between 0.005 Hz and ~200 Hz, higher frequency range can be achieved with a high-speed AFM. Compared to single-frequency oscillatory AFM measurements, the new AFM^2^ method offers a distinct advantage for rapid measurements of cell response at low frequencies (i.e., down to ~0.005 Hz within 30 seconds). This information allows us to discover a characteristic transition *T*_*low*_ in loss tangent (*G*″(*ω*)/*G*′(*ω*)) that can be used as a diagnostic tool to indicate the capability of cell restructuring. The observation sheds light on the cell migration properties of invasive cancer cells. We show that single cell migration (i.e. mutant PDAC p53^R172H^ cells) requires the ability to rapidly restructure the cytoskeleton, which corroborates with many previous observations^52^. In this regard, p53 mutation does not affect this capability, although it increases cell stiffness. However, in the case of collective migration (i.e. 4HT activated ROCK:ER cells), invasion will be facilitated by synchronised group movement. In this case, individual cells in a cluster will become softer and more fluid-like. These findings would not be possible if *G*′(*ω*) and *G*″(*ω*) were studied over a narrow and discrete frequency range and in isolation.

It is germane to note that several factors may have affected the implementation of the AFM^2^ method and led to atypical frequency dependence for *G*″(*ω*) response of cells (e.g. Fig. 3). These include indentation location and indentation depth, which we remind to be 400 nm above the nuclei area in this study. Since cell cortex in most cases is relatively thin^53^, it is likely that the stress-relaxation data contain contributions from the nuclei. This may account for the absence of power-law dependence in *G*″(*ω*) for PDAC p53^R172H^, PDAC p53fl/fl and GFP:ER cells (Figs 3–5), where cell height was ~3.5 μm and there was hardly any visible F-actin layer over the nuclei region (Fig. 6). This conclusion is corroborated with the phenomenon observed in the 4HT treated ROCK:ER cells. The treatment resulted in increased cell height (~8 μm high), absence of actin stress fibers in the cell and a thicker F-actin cytoskeleton over the nuclei area (Fig. 6), and consequently a typical weak power law dependence in agreement with previous works^8,31^. Furthermore, for the rounded cells on patterned substrates, the exact location of cell nucleus is difficult to determine, which introduces additional uncertainty to the measurement. Together, these results highlight the need of further studies, both experimentally and theoretically, to dissect the relative contribution of the nuclei and cytoskeleton to the whole cell viscoelasticity.

In summary, the AFM^2^ method provides a fast and powerful tool to correlate molecular–regulated cytoskeleton modulations with the global mechanical (i.e. viscoelastic) properties of living cells.

## Methods

### Materials

The complex materials used for experimentation include polydimethylsiloxane slabs (PDMS; 20:1 silicone elastomer to curing agent ratio; Dow Corning), and a 5% w/v polyacrylamide (18 × 10^6^ MW, Polysciences Inc., Warrington) gel-like solution (PAAM) in d.H_2_O. The mixture was left on a shaker plate for 1 week to ensure the monomer is homogeneously distributed throughout the solution. The PDMS samples were evenly mixed, degassed and baked for 2 hour at 70 °C. All materials were kept at room temperature before use.

### Cells and cell culture

The pancreatic ductal adenocarcinoma (PDAC) mutant p53 (denoted as PDAC p53^R172H^) and deleted p53 cell (denoted as PDAC p53^fl/fl^) lines were prepared as described previously^20^. PDAC p53^fl/fl^ cells were further modified by retroviral transduction and drug selection to create three cell lines^34^: (1) expressing conditionally active ROCK1- or ROCK2-estrogen receptor (ER) fusion proteins (denoted as ROCK1:ER and ROCK2:ER cells respectively, or ROCK:ER cells for both). (2) expressing green fluorescent protein-estrogen receptor fusion protein (denoted as GFP:ER cells), which are used as control. PDAC cell lines were cultured in DMEM supplemented with 10% FBS, 2 mmol/L L-glutamine, and penicillin-streptomycin (complete DMEM). To activate ROCK kinase activity, 1 μM of 4-hyroxytamoxifen (4HT) was added to the culture medium.

### Fabrication of cell patterns

To evaluate the influence of cell shape on their viscoelastic properties, micropatterned substrates were created to control cell morphology. The micropattern consisted of an array of 20 μm circles with 80 μm gaps, and was fabricated on glass slides as described previously^36^. The circular areas were modified with amino groups to promote cell adhesion, and the remaining areas were modified with polyethylene glycol (PEG) to prevent cell adhesion. Prior to cell seeding, the slides were sterilised with ethanol and phosphate buffered saline (PBS) for 10 minutes each, at room temperature. A cell density of ~1 × 10^5^ cells/cm^2^ were seeded onto the micropatterned substrates, and incubated for 2 hours at 37 °C, with 5% carbon dioxide (CO_2_). Once the majority of cells attached, unsuspended cells were washed away with culture medium. The cells on substrates were incubated for another 2 hours to enable cells to fully attach and spread^54^.

### Immunofluorescence staining

Cells were washed with PBS and fixed with 3.8% formaldehyde, 2% sucrose in PBS for 10 minutes at room temperature. The cells were permeabilised with 0.1% triton X-100 in PBS for 10 minutes at room temperature, and blocked with 1% bovine serum albumin (BSA) in PBS for 30 minutes. Actin filaments were stained using phalloidin in PBS with BSA (1:500; Alexa Fluor^®^ 488, Life Technologies) for 1 hour at room temperature. The stained cells were washed in 0.5% Tween 20 in PBS and mounted onto a coverslip using Vectashield mounting medium with DAPI (Vector Laboratories Inc.) or Bright field and fluorescence images were collected using a Zeiss AX10 microscope. For fluorescence images, excitation filters of 340 ± 10 nm (blue, DAPI) and 485 ± 10 nm (green, FITC) were used for the two stains.

### Atomic Force Microscopy measurements

An atomic force microscope (NanoWizard II) coupled with an inverted optical microscope (Zeiss AX10) was used in this study. Spherical AFM probes were made by gluing a 4.74 μm silica bead (Bang labs) onto a tipless ARROW-TL1 cantilever (k_nom_ = 0.06 N m^−1^, Nanoworld) or gluing a 20 μm silica bead (Bang labs) onto a tipless FORT-TL cantilever (k_nom_ = 0.6–3.7 N m^−1^, Nanoworld)^24^. The sensitivity and spring constant of each AFM probe was carried out before and after each experiment in air using the thermal noise method (JPK Manuel) to ensure the reliability of the measurements. For the beaded Arrow-TL1 cantilevers, these values were typically around ~90 nm V^−1^ and 0.05 N m^−1^. For the beaded FORT-TL cantilevers, they were typically 41.79 nm V^−1^ and 2.01 N m^−1^. The 1^st^ resonance peak for the beaded ARROW-TL1 cantilever in liquid is 1.0 kHz while that of the beaded FORT-TL cantilever is 24 kHz in liquid.

The complex materials were measured in liquid at room temperature. The cells were placed on a heater stage and measured in HEPES (4-(2-hydroxyethyl)-1-piperazineethanesulfonic acid) buffered media at 37 °C. Stress-relaxation experiments were carried out by indenting a sample to a pre-defined indentation depth and maintaining constant height with closed-loop feedback control in z-axis, as shown in Fig. 1a. Several loading rates (3–100 μm/s) were examined for indentation. For complex materials, 3 randomly chosen areas on a sample were measured. For cells, indentation position was placed above the centre of the nuclei area to avoid the artefacts from the nuclei boundary. The pre-defined indentation depth of ~400 nm was chosen to ensure the validity of the Hertz model and consistence of comparison^24^. Considering single cell heterogeneity, a conventional instantaneous nanoindentation was performed prior to the stress-relaxation test to identify the loading force for reaching the pre-defined ~400 nm indentation depth. For each condition, 50 randomly selected cells from throughout the culture area were probed in the above manner, error bars showing standard deviation.

### Bulk rheology

Oscillatory measurements were conducted with a modular compact rheometer, MCR 302 (Anton Paar) equipped with a parallel plate tool of 25 mm diameter (PP25; Anton Paar). Oscillatory shear measurements were performed over a range of angular frequency between 0.1 < *ω* < 100 rad/s. These were performed at room temperature.

### Statistics

All data are reported as mean ± 1 Standard deviation. Statistical analysis was carried out using GraphPad Prism 6 Software using two-way ANOVA and a post hoc Sidak test. The statistical significance cut off was set as p value < 0.05. A D’Agostino & Pearson omnibus normality test was carried out to determine population distribution, where normalised distribution has a p value > 0.05. Power law exponents were determined by a linear fit of the moduli drawn in a Log-Log plot. As the inflection point was very distinct, the identification of this was determined visually.

The datasets generated during and/or analysed during the current study are available from the corresponding author on reasonable request.

## Electronic supplementary material

Supplementary information
