# Supplementary material for: A one-step procedure to probe the viscoelastic properties of cells by Atomic Force Microscopy
Source: Sci Rep. 2018 Sep 27;8:14462. doi: 10.1038/s41598-018-32704-8 (PMC6160452; doi:10.1038/s41598-018-32704-8)
Supplement: Supplementary file 1 — Supplementary information [file 41598_2018_32704_MOESM1_ESM.docx]

A one-step procedure to probe the viscoelastic properties of cells by Atomic Force Microscopy

Ya Hua Chim^1^, Louise M. Mason,^1^ Nicola Rath^2^, Michael F. Olson^2^, Manlio Tassieri^1*^ & Huabing Yin^1*^

1 Division of Biomedical Enineering, School of Engineering, University of Glasgow, Glasgow G12 8LT, UK, 2 Cancer Research UK Beatson Institute, Garscube Estate, Switchback Road, Glasgow G61 1BD, UK.

Correspondence and requests for materials should be addressed to M.T. ([Manlio.Tassieri@glasgow.ac.uk](mailto:Manlio.Tassieri@glasgow.ac.uk)) and H.Yin ([huabing.yin@glasgow.ac.uk](mailto:huabing.yin@glasgow.ac.uk))

**Supplementary Information:**

**Figure S1. Raw stress-relaxation data for the curve shown in Figure 1.**

**Figure S2. The Front panel of the LabView executable software.**

**Figure S3. The transfer function of the Z Piezo used in this study.**

**Figure S4. The effect of approach ramp speeds.**

**Figure S5. Phase contrast and fluorescence images of PDAC p53^R172^ and PDAC p53^fl/fl^ cells.**

**Figure S6. The effect of retroviral transduction on complex moduli.**

**

**

**Figure S1.** Representative raw stress-relaxation data from 5% polyacrylamide gel-like solution (PAAM); the raw stress-relaxation curve used in the schematic shown in Figure 1. The curve was obtained on a 5% polyacrylamide gel-like solution using an ARROW-TL1 cantilever with an attached 4.7 μm silica bead. The insert shows the close-view of the force curve before indentation and after retraction. The slightly drift (<50pN) after the retraction is likely due to the substantial adhesion observed before separation causing the cantilever res-position to shift by a few nanometres.


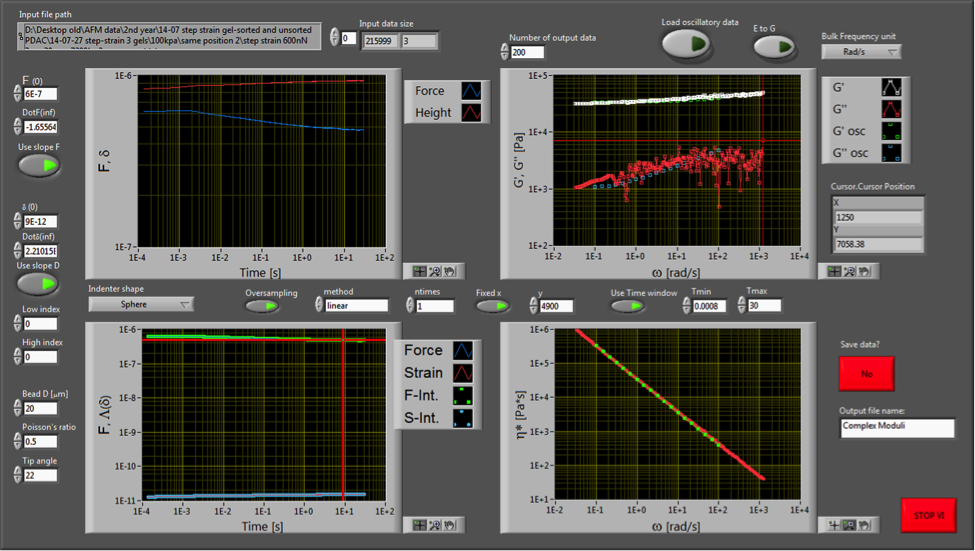


Figure S2. Front panel of the LabView executable developed to educe the materials’ linear viscoelastic properties from AFM indentation measurements. (Top-left graph) Input of raw relaxation force-indentation data (i.e. $\left[ \boldsymbol{t, F, \delta} \right]$) (Bottom-left graph) F(t) and Λ(δ(t)) based on Equation 2 to take into account the indenter’s geometry; (Top-right graph) The material’s storage (G’) and loss moduli (G”) over a continuous frequency spectrum; (Bottom-right graph) The material’s complex viscosity $\left| \boldsymbol{\eta}^{\boldsymbol{*}} \right|\boldsymbol{=}{\sqrt{\boldsymbol{G'}^{\boldsymbol{2}}\boldsymbol{+}\boldsymbol{G"}^{\boldsymbol{2}}}}/\boldsymbol{\omega}$.





Figure S3. The transfer function of the Z Piezo used in this study.


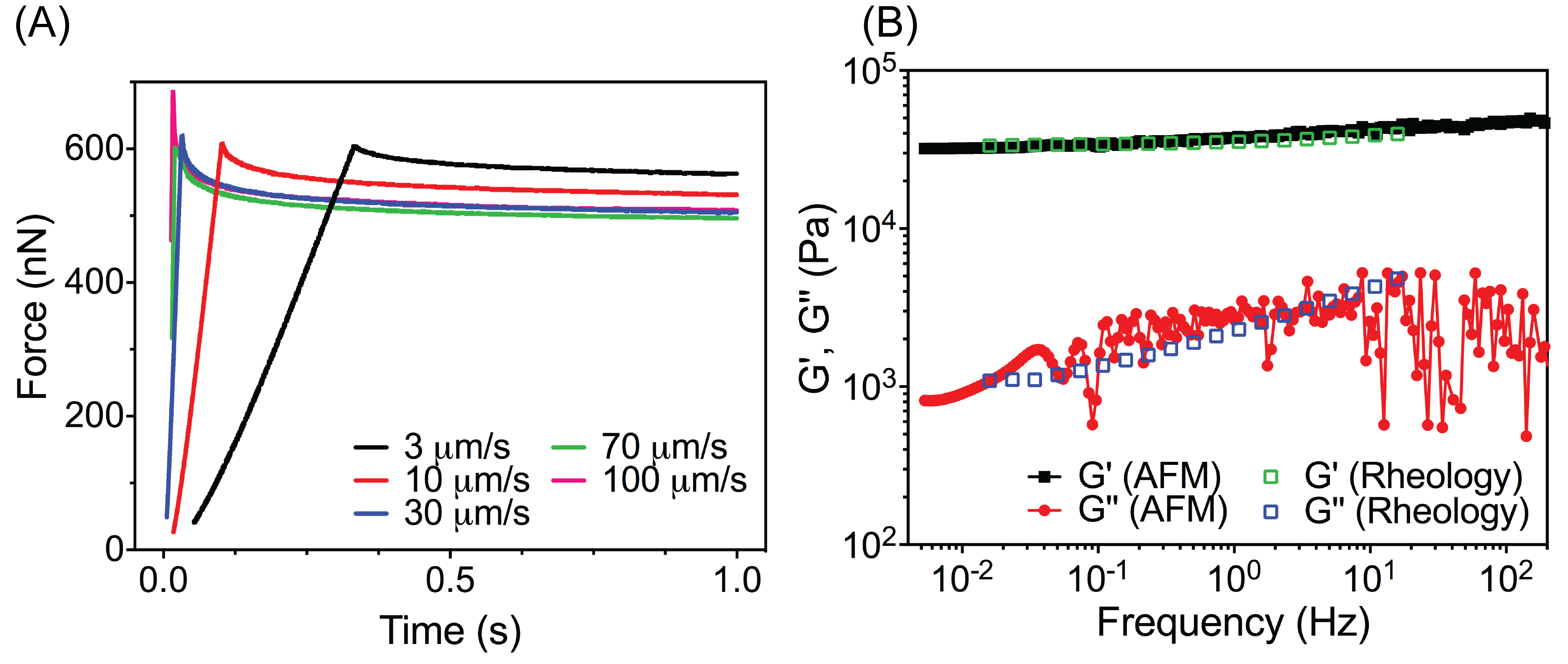


Figure S4. (A) The effect of approach ramp speeds. The force relaxation curves for a100 kPa gel (250 μm thick, ExCellness Biotech SA) showed that the final forces exceeded the pre-set values at speeds > 70 µm/s. (B) The viscoelastic moduli of the 100 kPa gel obtained using the AFM^2^ method at the indentation speed of 10 µm/s. The G’ and G” moduli measured using a traditional bulk rheometer (open symbols) were included for comparison.


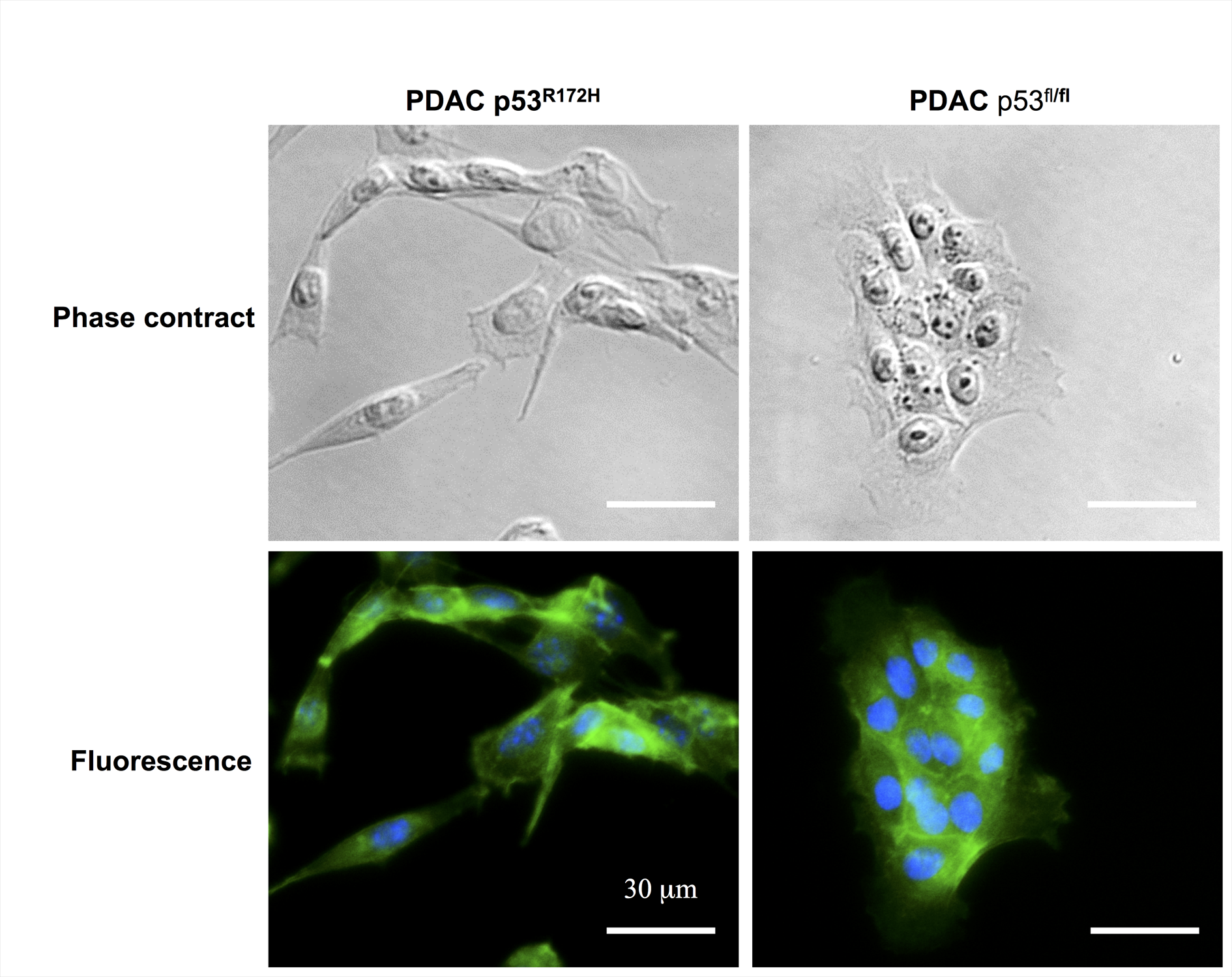


**Figure S5.** Phase contrast and fluorescence images of PDAC p53^R172^ and PDAC p53^fl/fl^ cells. PDAC p53^R172^ often migrate as individual cells whereas PDAC p53^fl/fl^ cells always form clusters.


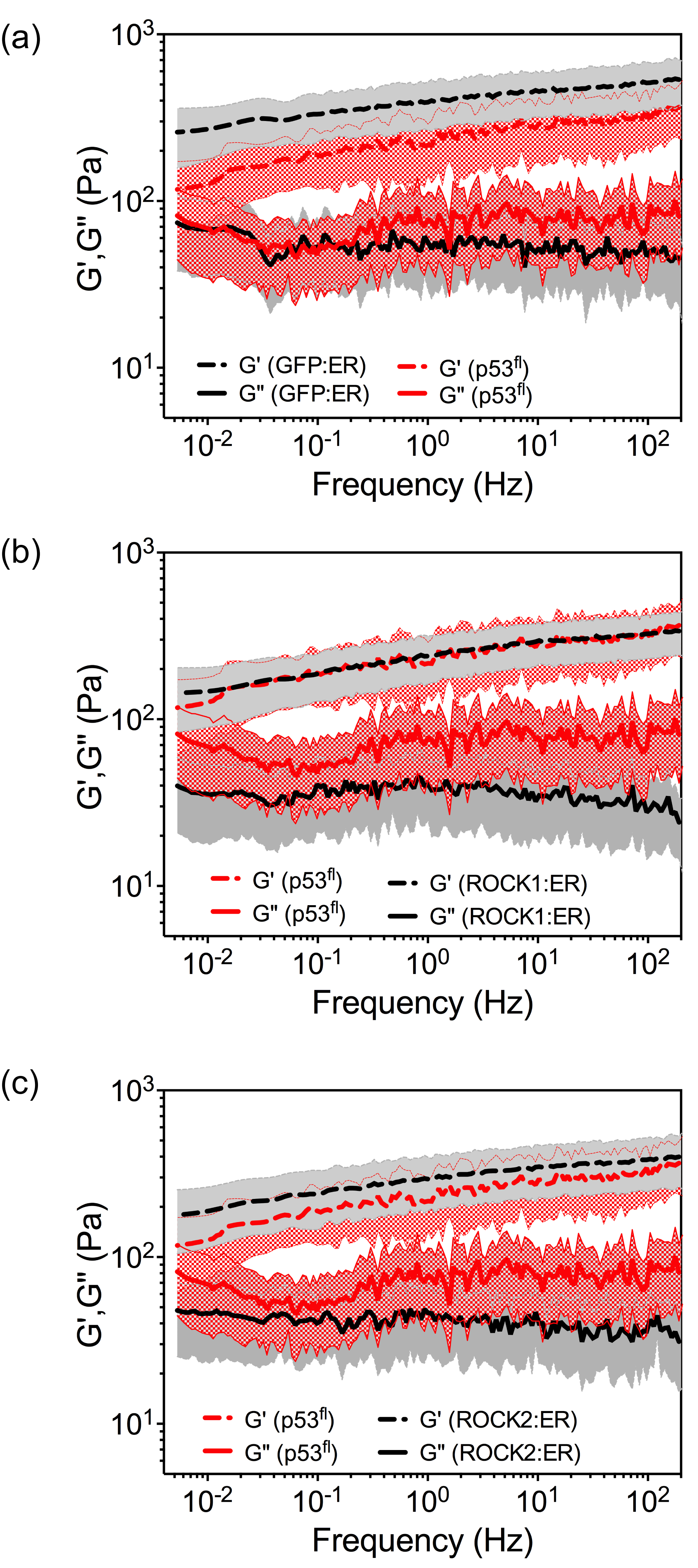


Figure S6. The effect of retroviral transduction on complex moduli ($\boldsymbol{G'}\left( \boldsymbol{\omega} \right)$, $\boldsymbol{G''}\left( \boldsymbol{\omega} \right)$). Parental PDAC p53^fl^ cells were modified by retroviral transduction to create ROCK1:ER, ROCK2:ER and GFP:ER cells.
